# What influences the implementation of kangaroo mother care? An umbrella review

**DOI:** 10.1186/s12884-022-05163-3

**Published:** 2022-11-18

**Authors:** Qian Cai, Dan-Qi Chen, Hua Wang, Yue Zhang, Rui Yang, Wen-Li Xu, Xin-Fen Xu

**Affiliations:** 1 Nursing Department, Zhejiang University School of Medicine, Hangzhou, Zhejiang China; 2 Zhejiang University School of Medicine Women’s Hospital, No. 1 Xueshi Road, Shangcheng District, Hangzhou, 310006 Zhejiang Province China; 3 Women’s Hospital School of Medicine Zhejiang University Haining Branch/Haining Maternity and Child Health Care Hospital, No. 6 Qinjian Road, Haizhou Street, Haining, 314400 Zhejiang Province China

**Keywords:** Kangaroo mother care, Preterm birth, Umbrella review, Implementation, Barriers, Facilitators

## Abstract

**Background:**

Kangaroo mother care (KMC) is an evidence-based intervention that reduces morbidity and mortality in preterm infants. However, it has not yet been fully integrated into health systems around the world. The aim of this study is to provide a cogent summary of the evidence base of the key barriers and facilitators to implementing KMC.

**Methods:**

An umbrella review of existing reviews on KMC was adopted to identify systematic and scoping reviews that analysed data from primary studies. Electronic English databases, including PubMed, Embase, CINAHL and Cochrane Library, and three Chinese databases were searched from inception to 1 July 2022. Studies were included if they performed a review of barriers and facilitators to KMC. Quality assessment of the retrieved reviews was performed by at least two reviewers independently using the Joanna Briggs Institute (JBI) critical appraisal checklist and risk of bias was assessed with the Risk of Bias Assessment Tool for Systematic Reviews (ROBIS) tool. This umbrella review protocol was documented in the PROSPERO registry (CRD42022327994).

**Results:**

We generated 531 studies, and after the removal of duplicates and ineligible studies, six eligible reviews were included in the analysis. The five themes identified were environmental factors, professional factors, parent/family factors, access factors, and cultural factors, and the factors under each theme were divided into barriers or facilitators depending on the specific features of a given scenario.

**Conclusions:**

Support from facility management and leadership and well-trained medical staff are of great significance to the successful integration of KMC into daily medical practice, while the parents of preterm infants and other family members should be educated and encouraged in KMC practice. Further research is needed to propose strategies and develop models for implementing KMC.

**Supplementary Information:**

The online version contains supplementary material available at 10.1186/s12884-022-05163-3.

## Background

According to reports from the World Health Organization (WHO), with the development of assisted reproductive technology and the improvement of emergency and critical care technology, the incidence of premature birth is rising, and premature birth has become a global problem [1]. Nearly fifteen million preterm infants are born each year, and more than one million of them unfortunately die each year [2]. According to statistics, complications of preterm birth directly account for more than 35% of all neonatal deaths, while the proportion of deaths indirectly caused by preterm birth is even higher because preterm birth increases the risk of infant death from infection [3]. Many surviving preterm infants encounter plenty of problems due to premature birth, such as sensory impairment and cognitive and language impairment [4–6]. In addition, the birth of preterm infants may cause a substantial emotional crisis and economic cost to the family, as well as have an impact on public sector services such as education and other social support systems [7, 8]. For mothers, preterm birth may also cause a range of perinatal diseases [5, 9]. Therefore, effective evidence-based interventions that can be implemented at scale are urgently needed to reduce the incidence of preterm birth complications and neonatal mortality.

Kangaroo mother care (KMC) is one such evidence-based life-saving intervention for preterm infants [10]. In KMC, the mother (or father) puts her (his) naked preterm infant on her (his) chest in the same way as kangaroo parenting so that the preterm infant is capable of having early, continuous and long-term skin-to-skin contact with his or her mother (father); in addition, measures such as exclusive breastfeeding or breastfeeding, early discharge, and follow-up after discharge are taken for the preterm infants [11, 12]. Compared with the conventional nursing mode, KMC is not only able to maintain the body temperature of preterm infants but also significantly reduces the risk of death in low-birth-weight infants by 36% while significantly reducing the risk of sepsis, hypoglycaemia, and hypothermia [13]. Numerous studies have shown that KMC is a safe, effective, and multifaceted intervention with many short-term and long-term positive effects for preterm infants, such as stabilizing the neonatal physiological state, enhancing immunity, increasing exclusive breastfeeding rates, and promoting mother-infant bonding [14–17].

Despite the clear benefits of KMC, this intervention has not yet been fully integrated into health systems around the world [18, 19]. There are many barriers impeding the implementation of the KMC, including but not limited to lack of support from family members, lack of parental information, and lack of tools and resources [20–23]. Several studies have identified facilitators that may contribute to the implementation of KMC, such as providing KMC training programmes for parents and encouraging physicians to recommend KMC to parents [24–26]. Undoubtedly, a better understanding of these barriers and facilitators can optimize the implementation of KMC.

Studies on the subject of KMC have developed over many years, with extensive studies from around the world and several systematic reviews on KMC published. These studies spanned different clinical settings, and there are studies that have explored the influencing factors of KMC from different perspectives, such as caregivers (e.g., parents and families) and healthcare workers [27–29]. A certain number of barriers and facilitators have been identified in these studies. However, the complexity and diversity of conventional studies make KMC difficult to describe and understand and impose challenges for health professionals and administrators who try to apply KMC in health systems [22, 30]. Therefore, it is necessary to robustly summarize the evidence base to identify and elucidate key barriers and facilitators to the implementation of KMC.

One available approach is the umbrella review, which involves the synthesis of existing reviews, enabling researchers to collect evidence from multiple healthcare facilities instead of conducting systematic reviews at each facility. Essentially, an umbrella review is a review of existing reviews to provide an overview of the available evidence on a specific topic and allow comparisons of published reviews [31]. Furthermore, an umbrella review is capable of compiling evidence bases related to specific issues in a relatively short time frame [32]. We adopted this comprehensive assessment approach to outline factors that may facilitate or inhibit KMC implementation and expansion.

## Methods

### Protocol and registration

A protocol was prospectively developed in accordance with the Preferred Reporting Items for Systematic Review and Meta-Analysis Protocols (PRISMA-P) guidelines [33]. Following current recommendations, the protocol was made openly available through registration with the PROSPERO International Prospective Register of Systematic Reviews platform (registration number CRD42022327994).

### Study design

This review was conducted according to the rules for conducting umbrella reviews and published approach [32, 34], and was reported following the Preferred Reporting Items for Systematic Review and Meta-Analysis (PRISMA 2020) statement [35]. The PRISMA checklist is shown in Additional file 1.

### Search strategy

Electronic databases, including PubMed, the Cochrane Database of Systematic Reviews, EMBASE, the Cumulative Index to Nursing and Allied Health Literature (CINAHL), the China National Knowledge Infrastructure (CNKI, for Chinese literature), SinoMed (for Chinese literature), and WAN FANG DATA (for Chinese literature), were searched to identify systematic reviews and meta-analyses (published from database inception to 1 July 2022.) of the factors influencing the implementation of KMC in preterm infants. Additionally, we manually searched reference lists from the screened articles to avoid the omission of any related articles. Also, we searched Google Scholar and OpenGrey for grey literature.

The search terms were constructed by combining subject terms and free words, while the language was limited to Chinese or English. The English search terms used were “prematur*/preterm*/premie*/neonat*/infant*/newborn*/low birth weight/LBW/ NICU”, “kangaroo mother care/kangaroo mother method/kangaroo care/kangaroo attachment/kangaroo contact/KMC/KC/skin-to-skin care/skin-to-skin contact/SSC/mother-infant contact”, and “systematic review/meta-analys”, and “早产儿/新生儿/低出生体重儿”“袋鼠护理/袋鼠式护理/皮肤接触”“系统评价/Meta分析/荟萃分析” were adopted as the Chinese search terms. More details of the search strategies are shown in Additional file 2.

### Inclusion criteria

This umbrella review included studies published in peer-reviewed journals and grey literature that addressed the research question. Articles were included if they were published in Chinese, English or in other language with the English version; identified factors impacting KMC implementation, including barriers and facilitators as primary or secondary objectives; and were a systematic review or meta-analysis. Moreover, to retrieve valuable information about the subject under study, we also decided to include scoping reviews, a type of review study that uses a systematic method of searching for information with the aim of accumulating as much evidence as possible and mapping the results. Screening of the searched articles and their subsequent full-text review were carried out based on the following inclusion criteria: (a) studies that used a systematic/scoping review and/or meta-analysis design, (b) studies focused on preterm infants with KMC, and (c) studies that aimed to identify factors associated with KMC implementation. In addition, articles fulfilling the following criteria were excluded: (a) reviews written in any language other than English or Chinese, (b) duplicate publications, and (c) articles or conference abstracts for which the full text was not available.

### Study selection

Two researchers independently screened the literature according to the inclusion and exclusion criteria. In case of disagreement, the two researchers first discussed and attempted to resolve the disagreement. If the disagreement could not be resolved, a third researcher was invited to adjudicate. The literature screening process was as follows: (1) Endnote (a literature management software) was used to remove duplicate records; (2) the title and abstract of the articles were read in Endnote, and those that were not related to the subject, population and literature type were removed; (3) the full text of the remaining articles was downloaded, excluding those for which the full text could not be obtained; and (4) the full texts of the articles were read to further exclude literature according to the standard cited in the second step. The study selection process is summarized in the Preferred Reporting Items for Systematic Reviews and Meta-Analyses (PRISMA) flow diagram.

### Quality assessment

The quality of the included reviews was assessed using the Joanna Briggs Institute (JBI) critical appraisal checklist for systematic reviews and research syntheses [36]. This assessment tool comprises 11 items, and the evaluation criteria for each item are “yes”, “no”, “unclear” or “not applicable”. Two members independently assessed the retrieved articles. Any disagreement between them was resolved by a third investigator.

### Risk of bias assessment

Risk of bias of the included studies was evaluated by two reviewers using the Risk of Bias Assessment Tool for Systematic Reviews (ROBIS) [37]. In case of disagreement, a third reviewer was consulted until a final decision was made. ROBIS assesses four domains: 1) study eligibility criteria; 2) identification and selection of studies; 3) data collection and study appraisal; and 4) synthesis and findings. Each domain consists of five to six questions with six possible options: Yes, Probably yes, Probably No, No, Not indicated or Not applicable.

### Data extraction

Two researchers independently used a unified Excel form that served as a data extraction sheet used to extract variables that were relevant to the scope of the current review, and another researcher verified the accuracy of the data extraction and quality assessment of all the included reviews. The extracted variables included the type of review, years covered, the total number of studies included in the review, country of origin, settings, aims/objectives and participants. As the aim was to provide a broad overview, all barriers and facilitators in all of the reviews were extracted except for those that were infrequently reported (i.e., those reported by only a few studies).

### Data synthesis

After the data were extracted, a qualitative content analysis of the factors impacting KMC implementation was undertaken by the researcher. Each review article was read carefully to identify and extract the reported barriers and facilitators, and the researcher prepared the tables to summarize the data of all articles (see Additional file 3). The main key factors extracted from the articles were grouped and classified into themes to enhance the comprehension of the results outcomes. This classification of findings was performed based on the identified factors from the studies included in this review. Any uncertainties regarding the thematic categorizations were resolved through discussion and consensus by the reviewers.

## Results

**Fig. 1:**
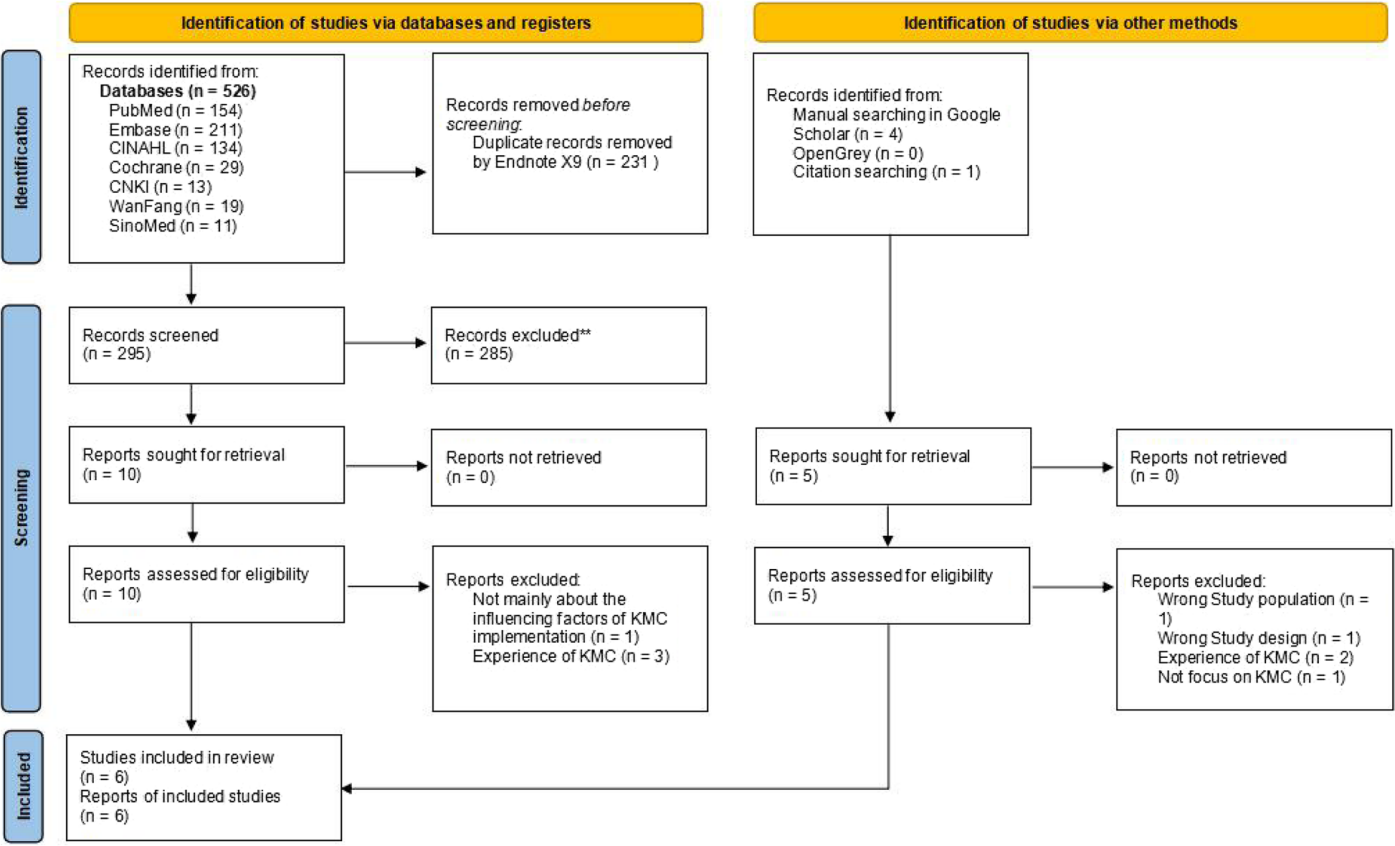
PRISMA flow diagram of barriers and facilitators to implementing KMC

Five hundred and thirty one hits retrieved in the initial search were exported into the reference management software Endnote, and 300 of them was left after duplicate records were excluded. A total of 285 references whose subject and theme were not matched were removed after title and abstract screening. Six eligible reviews were included after further full-text screening of the remaining 15 articles, as shown in Fig. 1.

### Study characteristics

Table 1 provides an overview of five systematic reviews and one scope review related to KMC implementation as of July 1, 2022, all of which were published in 2015 and later, indicating this topic is relatively fresh. Two of the six articles described barriers and facilitators of KMC implementation from the perspective of caregivers of preterm infants [27, 39]; one article explored these influencing factors from the the perspective of healthcare workers [28]; and the remaining articles discussed the factors affecting KMC implementation from both the perspectives of healthcare workers and parents of preterm infants [29, 38, 40].

**Table 1:** Characteristic of included reviews

Author/year	Type of Review	Years covered	Number of studies in the review	Country	Setting	Aim/Objective	Participants
Smith et al. (2017) [27]	Systematic review	January 1, 1960 to August 19, 2015	98	Americas, Africa, Europe, Southeast Asia, Eastern Mediterranean, Western Pacific, and multiple regions	Health facility, NICU or stepdown unit, Community or population-based surveillance	To identify barriers and enablers of implementation and scale up of KMC from caregivers’ perspective.	Caregivers (e.g. mothers, fathers, and families) perspective
Chan et al. (2017) [28]	Systematic review	January 1, 1960 to August 19, 2015	86	Americas, Africa, Europe, Southeast Asia, Eastern Mediterranean, Western Pacific, and multiple regions	Health facility, NICU or stepdown unit, Community or population-based surveillance	To further explore the barriers and enablers of KMC implementation specifically from the perspective of health systems, with a focus on HCWs and health facilities	Perspective of healthcare workers (HCWs) and/or facilities
Seidman et al. (2015) [38]	Systematic review	Before August 13, 2013	103	Sweden, United States, Sub-Saharan Africa, South Africa, North Africa/the Middle East, Latin America/Caribbean, Eastern Europe, East Asia/ Southeast Asia/Pacific and multiple regions	Health facility, community-initiated setting	To identify the most frequently reported barriers to KMC practice for mothers, fathers, and health practitioners, as well as the most frequently reported enablers to practice for mothers.	Mothers, fathers, and health practitioners
Kinshella et al. (2021) [29]	Systematic review	From database inception to December 2019	30	Sub-Saharan Africa (Ghana, Nigeria, Malawi, Mozambique, South Africa, Zambia, Zimbabwe, Ethiopia, Tanzania, Uganda)	Health facilities in sub-Saharan Africa	To understand the barriers and facilitators of kangaroo mother care implementation in health facilities in sub-Saharan Africa, where there are the highest rates of neonatal mortality in the world	Health worker, mothers and their families
Mathias et al. (2021) [39]	Scoping review	January 1990 to August 2020	22	Low-and middle income countries (Bangladesh, Brazil, Ethiopia, Ghana, India, Indonesia, Malawi, Mozambique, Nigeria, Pakistan and South Africa)	Health facility, community-based surveillance	To map evidence on the barriers, challenges and facilitators of KMC utilisation by parents of LBWIs (parent of low birthweight infant [PLBWI]) in LMICs.	Parents of low birthweight infant
Chan et al. (2016) [40]	Systematic review	January 1, 1960 to 19 August, 2015	112	Americas, African, European, South-East Asia, Eastern Mediterranean, Western Pacific and Multiple regions	Health facility, NICU or stepdown unit, Community or population-based surveillance	To understand factors influencing adoption of kangaroo mother care in different contexts	Mothers, fathers and families; health-care workers and facilities

The number of studies included in each review varied significantly, which often depended on the inclusion scope of the review [27–29, 38–40]. For instance, two most recently published reviews included a smaller number of studies as it defined a specific study area [29, 39]. Most of the studies included in the reviews were carried out in low-and middle-income counties and were conducted in health facility.

### Quality assessment

The methodological quality of the included 6 articles was evaluated by the JBI critical appraisal checklist. The ninth item “Was the likelihood of publication bias assessed” for all the included articles was “No” because publication bias are not assessed in all the included reviews. As the tools for evaluating the quality of the included studies and how to evaluate the quality of the included studies were not described in the two studies conducted by Seidman et al. [38] and Mathias et al. [39], so the fifth item “Were the criteria for appraising studies appropriate” and the sixth item “Was critical appraisal conducted by two or more reviewers independently” for these two studied was “No”, and the evaluation results of the remaining items were all “Yes”. The results of the quality appraisal of all the included studies are displayed in Additional file 4.

### Risk of bias assessment

After applying the ROBIS tool for risk of bias evaluation, of the six included systematic reviews, four were evaluated to have a high bias risk [27, 28, 38, 40], and two present an unclear bias risk [29, 39] (see Additional file 5). Main concerns regarding this aspect were related to (a) limiting searches with language restrictions; (b) lack of risk of bias evaluation; and (c) selection and data extraction not done in duplicate.

### Barriers and facilitators of KMC

The five themes identified were environmental factors, professional factors, parent/family factors, access factors, and cultural factors. The subfactors under each theme were divided into barriers or facilitators according to the descriptions provided in the included reviews. A brief summary of the barriers and facilitators identified under each theme is presented in Table 2. These are described in more detail below.

**Table 2:** Summary of barriers and facilitators

Themes	Barriers	Facilitators
**Environmental Factors**	**• Facility conditions** Lack of privacy Insufficient space and supplies Temperature Issues with clothing / infants’ medical devices Logistical issues related to implementing new practice **• Resources and Materials** Lack of necessary resources Lack of KMC guidelines or protocols No checklist for KMC admission procedures Lack of electronic medical records for KMC Poor management of resources donated to the hospital KMC was not budgeted for, and resources were mismanaged Facilities did not provide food for mothers **• Healthcare system** Visitation policies were difficult KMC training not part of a broader healthcare training curriculum Inadequate/inconsistent training Unsupportive staffing policies Poor supportive supervision and record-keeping Inconsistent application of KMC ˙ Inconsistent application of KMC within facilities and among HCWs ˙ Inconsistent knowledge and application of kangaroo mother care Follow-up and discharge procedures not well structured Many facilities reported performing continuous KMC, but few actually practiced it Receiving visitors Only low birthweight infants received kangaroo mother care in some locations	**• Facility conditions** Access to private space/ privacy screens Sufficient space and supplies Temperature stability KMC ward Quiet and relaxed atmosphere **• Resources and Materials** Access to structural resources ˙ Use of technology ˙ Use of KMC expert clients ˙ Site assessment tools Use of KMC guidelines or protocols Displayed KMC pictures/posters Reporting and data Management mobilization of resources Breast milk banks provide milk and can be an educational tool among mothers Recreation activities **• Healthcare system** Integration of kangaroo mother care into health-care curriculum ˙ Expanding training to other healthcare personnel besides nurses Ongoing KMC education Supportive staffing policies Supportive Supervision and dedicated registers KMC policies Follow-up at the facility-based KMC Include KMC in health facility statistics into maternal health services Integrating KMC Use of performance standards and quality improvement measures
**Professional Factors**	**• Professional perception** Lack of belief in efficacy or importance ˙ Nurses believe KMC based on perception and not scientific fact ˙ Nurses fail to have strong belief in importance of kangaroo mother care KMC perceived not safe and causes infection and neck deformity Disagreement over clinical stability ˙ Medical stabilisation of LBWI perceived as restriction to KMC initiation Considered parents or visitors as an obstacle Concerns about other medical conditions / care Belief that KMC causes extra work Concerns about parents’ ability to practice **• Professional characteristics** Limited communication between HCWs Level of experience Lack of change mindset Unsupportive, loud, uncaring Inadequate knowledge Nurses not given feedback on kangaroo mother care data collected **• Professional Management** support Lack of leadership and management High staff and leadership turnover Management did not prioritize kangaroo mother care Management reluctance to allocate space for SSC Handoff issues with other nurses Need for high-touch support from staff	**• Professional perception** Believing KMC benefits ˙ Nurses were more likely to perform KMC if they believed it worked ˙ Nurses more likely to use kangaroo mother care after seeing positive effects **• Professional characteristics** Good communication Experience with KMC Staff acceptability and enthusiasm Nurses’ willingness to educate PLBWIs **• Professional Management** Leadership and management support Nurse involvement in care related decision making Multiple health worker support facilitated SSC -- nutrition workers, CHWs and clinical workers Practicing securing catheters lowered nurses’ concerns Mentorship and opportunities to share knowledge Availability of skilled KMC health workers KMC support groups facilitated KMC utilisation Management promotion of kangaroo mother care
**Parents/Family Factors**	**• Perception and Motivation** Experienced and perceived discomforts to the parent and/or LBWI associated with KMC ˙ Discomfort / unease with the situation Were unaware of the benefits of KMC Lack of awareness of KMC Perceived newborn did not enjoy KMC KMC felt forced ˙ Were expected to perform KMC with little or no instruction ˙ Could not see newborn during KMC ˙ Did not feel a bond with the infant ˙ Fears and discomforts with KMC practice Isolation effect ˙ Mothers lonely and depressed in KMC ward Negative impressions of staff attitudes or interactions Fear / anxiety of hurting the infant Felt less of women for having LBWIs Maternal attitude towards KMC PLBWI ridiculed by the family and community **• Parenting Capacity** Pain / fatigue ˙ Pain hindered KMC, particularly after a C-section Mother’s medical issues / post-partum depression Low self-esteem and lack of confidence Lack of knowledge on KMC Positioning issues (including sleeping) Breastmilk expression and others BF-related issues Demographics of mother or infant **• Support and empowerment** Lack of family support ˙ Mothers-in-law and grandmothers did not approve ˙ Family attitudes Staffing support (support from medical staff) ˙ Poor support or negative interactions with medical staff ˙ HCWs Did not respect family privacy Disapproval from community P to perform KMCdesireeer pressure negatively influenced Lack of help with KMC practice and other obligations General lack of buy-in / low perceived value Disempowerment in decision-making	**• Perception and Motivation** Perceived and experienced KMC benefits ˙ Newborns slept longer, less anxious, happier, more willing to feed ˙ KMC was calming, relaxing, comforting, natural, instinctive, secure, logical, healing ˙ Created a family bond, inspired caregiver confidence ˙ Sped emotional and physical recovery of mother ˙ Made caregivers feel useful ˙ Mother-infant attachment ˙ Calming, natural, instinctive, healing for parents and infant Understanding of efficacy / benefits KMC awareness Belief that infant enjoys practice Feelings of confidence / empowerment Ease of practice / preference over traditional care Early discharge as motivator Positive attitudes toward PT survival **• Parenting Capacity** Health condition ˙ KMC helped mother’s recover from post-partum depression ˙ Managing postpartum pains Maternal confidence/will to practice KMC KMC knowledge Ability to stay with infant Health seeking behaviour **• Support and empowerment** Family support ˙ Grandmothers, sisters, others helping with chores increased uptake and duration of KMC ˙ Paternal support crucial to success of KMC, they alleviate workload, support, encourage, increase mother’s confidence ˙ Family more likely to understand and respond well if mother explained KMC ˙ Improved family interactions Staffing support (support from medical staff) ˙ Support from staff or community health worker (CHW) ˙ Access to staff and training on KMC ˙ Receiving support from medical staff ˙ Good nurse -- mother relationship Community support with KMC practice Peer support from other mothers Support from government Incorporating mothers in decision making on LBWIs’ care Empowerment in decision-making Continuous training and support ˙ Return demonstration
**Access Factors**	**• Time / Workload** Limited visitation time ˙ Shortage of staff nurses limited parental access and shortened visitation time ˙ The shorter the visitation period was, the more of an interference staff thought parents were Actual increased workload / staff shortages Takes away time from other patients ˙ Training mothers to do SSC would take additional time out of health workers’ schedules, increase their workload, and reduce time with other critical patients ˙ Health-care workers has difficulty finding time for training Caregivers unable to devote time ˙ Time needed to commute from home to hospital was too much ˙ KMC consumes time for house chores ˙ Stresses related to extended hospitalization The season of the year (Season in which the mother delivered) **• Location** Other responsibilities at home or work interfered Home delivery: late/delayed KMC initiation **• Financing** Cost associated with travel, food, lodging, parking, clinical fees ˙ Lack of money for transportation, beds and kangaroo mother care wrappers Difficulty accessing facility ˙ Lack of transport and distance to facility	**• Time / Workload** Unlimited visitation hours at health facility Kangaroo mother care did not increase workload Some nurses reported that KMC did not increase the amount of time they spent on each patient Early KMC initiation **• Location** Parents preferred to practice KMC at home than at the facility to at tend to other responsibilities ˙ Kangaroo mother care at home allowed parents to perform other duties Hospital delivery: prompt KMC uptake **• Financing** Lowering hospital costs to families ˙ Belief that KMC cut down hospital bills due to early discharge ˙ Belief that kangaroo mother care was cheaper than incubator care Lower costs for health system Parents more likely to stay if services were free
**Cultural Factors**	**• Traditional newborn care** Traditional bathing, carrying and breastfeeding practices did not always align with kangaroo mother care guidelines ˙ Bathing practices interfered ˙ Infants traditionally carried on back, thus carrying on the front seemed odd If breast feeding not pursued KMC less likely to continue Bathing practices and wrapping infants soon after birth delayed SSC Type of wrap: traditional chitenje **• Traditional mindset** Country or culture-specific beliefs, practices, or policies ˙ Cultural association of infants skin rash to mother-infant skin contact ˙ Cultural/traditional belief of waiting for the umbilical cord to fall off before KMC started Stigma and shame ˙ Mothers reported shame of having a preterm infant ˙ Fear, guilt doing KMC publically Considered unclean where diapers not used In warm climates staff did not believe hat and socks were necessary KMC hinders social obligations KMC considered as taboo **• Gender Roles** Felt KMC was role of mother Fathers lack of opportunity to practice ˙ Mothers did not want father to perform KMC ˙ Nurse excluding father from infant care was a cultural norm ˙ The males not allowed in the KMC room Lack of male involvement	**• Traditional newborn care** Some HCWs advised mothers to delay bathing so infant would not get cold Type of wrap: customised **• Traditional mindset** Country-specific beliefs or practices Mother-infant confinement **• Gender Roles** Gender equality Societal acceptance of paternal involvement Normalization of paternal involved in child care Male involvement

#### Environmental factors

This theme comprised facility conditions, resources and materials, and the healthcare system. Facility conditions mainly refer to hardware support in medical institutions, the most common factors being space and privacy. Lack of privacy and insufficient space and supplies directly hinder the implementation of KMC [27–29, 38–40], while access to private space/privacy screens and sufficient space and supplies are key facilitators for the implementation of KMC [27–29, 40]. In addition, factors such as temperature stability and a quiet and relaxed atmosphere in clinical facilities are conducive to the implementation of KMC [27, 28, 40]. Resources and materials refer to the environmental software support mainly related to resource management and material access. The most common barrier is a lack of KMC guidelines or protocols in the clinical unit [27–29, 38], while the implementation of KMC would be enhanced if the clinical unit adopted KMC guidelines or protocols and displayed KMC pictures/posters, etc. [28, 29, 39]. The healthcare system mainly involves educational and policy factors. Inadequate/inconsistent training and unsupportive staffing policies are barriers to KMC implementation [28, 29, 39, 40], while the integration of KMC into the healthcare curriculum and KMC-related policies are important facilitators for KMC implementation [29, 40].

#### Professional factors

This theme encompassed three subthemes: professional perception, professional characteristics, and professional management. The main barriers under this theme included medical staff’s lack of belief in the efficacy or importance of the KMC [38, 40] and their perceptions that KMC is unsafe [28, 39] and imposes extra workload on them [38], the limited level of experience and knowledge of health care workers [28, 29, 38] and lack of communication with each other [28], high staff and leadership turnover [28, 40] and lack of leadership and management support [28, 38, 40]. The main facilitators under this theme included medical staff’s belief in KMC benefits [28, 29, 40] and their sufficient experience, passion, and willingness to implement KMC [28, 29, 39]; leadership and management support [29, 40]; and multiple health worker support [28, 39].

#### Parent/family factors

This theme involved parental perception and motivation, parenting capacity, and parental support and empowerment. Experienced and perceived discomfort [29, 39], a lack of awareness of the benefits of KMC [27, 29, 38, 39], and fear/anxiety of hurting the infant [38] were the most frequently identified barriers to the implementation of KMC. Parenting capacity mainly refers to the health state of the parents of preterm infants. Medical issues such as pain/fatigue [27, 38, 40] and postpartum depression [27, 29, 38] and lack of confidence and knowledge on KMC [39] were the most common barriers. Support and empowerment refer to the availability of support from family members [27, 29, 38–40], medical staff [28, 29, 38–40], community [39, 40], and peers [28, 29], which facilitates the implementation of KMC and hinders implementation otherwise.

#### Access factors

This theme involved time, location, and financing. For medical staff, time was a key barrier; staff perceived that the implementation of KMC would increase their workload [28, 29, 38, 40] and reduce time with other critical patients [28, 40], and they had difficulty finding time for training [40]. For the parents of preterm infants, commuting from home and the medical unit was another barrier that caregivers were unable to devote sufficient time in KMC practice due to long commutes [27] or dealing with heavy household chores [39]. The costs of transportation, accommodation renting, and KMC implementation in the clinical ward were the immediate challenges [27, 38, 40]. Lower hospital costs to family [27, 29, 40], lower cost for health system [29] and unlimited visitation hours [27, 28, 40] were conducive to the implementation of KMC.

#### Cultural factors

This theme comprised traditional newborn care, traditional mindset, and gender roles. Traditional newborn care approaches, such as traditional bathing, carrying and breastfeeding practices [27, 28, 40], and the type of wrap [39] were identified as barriers to the implementation of KMC. However, some aspects of newborn care facilitated the implementation of KMC, i.e., advising mothers to delay bathing [28]. Some mindsets such as feeling ashamed of having a preterm infant [27, 38, 40], believing that skin-to-skin contact between the preterm infants and their caregivers was inappropriate [29, 38, 39] and considering KMC to be taboo [39] were identified barriers to the KMC implementation. Additionally, gender inequality existing in the division of labour between fathers and mothers [27, 38] was not conducive to the implementation of KMC that KMC was regarded as a role responsibility of the mother, and the father was not allowed to participate in KMC [38–40] .

## Discussion

Our umbrella review highlighted different factors, each factor comprising barriers and facilitators, that influence the implementation of KMC, provide decision-makers in healthcare with an overview of the field and provide information for the implementation of KMC. All of the included reviews were published in 2015 or later, which confirms the growth and interest in the field of KMC. However, there is considerable heterogeneity in the evidence base on KMC, which makes translation into practice challenging.

Factors related to facility conditions, mainly including lack of privacy and insufficient space and supplies, were mentioned in all six included reviews, which might be related to the operation characteristics of KMC. Skin-to-skin contact is the most important part of the KMC procedure, which requires parents to undress their upper bodies and put their preterm infants on their chests, which is why a suitable physical environment is of great significance [11, 12]. Studies have reported that mothers felt uncomfortable and exposed due to the continuous coming and going of medical staff during KMC when insufficient KMC private space was provided, which has proved to be a serious barrier affecting the implementation of KMC in many countries around the world [41–43]. Therefore, medical units should strive to provide enough quiet, comfortable, and private space for NICUs to implement KMC. Apart from physical facility conditions, resources and materials were another factor. Limited by facility space and human resources, some hospitals in China had to perform intermittent KMC instead of continuous KMC [44]. A multicountry analysis of health system bottlenecks from 12 African and Asian countries reported that insufficient essential supplies in facilities to support KMC was a barrier to the implementation of KMC [21].

KMC should be systematically implemented within a facility in accordance with relevant rules and regulations, for example, by adopting standard checklists for mothers and infants to ensure orderly and standardized KMC implementation. In a majority of the hospitals, nurses were required to commit to KMC-related tasks such as KMC recording, assessment, and data monitoring due to the lack of relevant rules and regulations, which meant an extra workload for the nurses [45, 46]. Studies have shown that human resource challenges, record keeping, and data collection are barriers to KMC implementation in countries such as Malawi and Indonesia [28, 47]. Documentation and annotation of KMC implementation were still not common practices in NICUs, while KMC-related information was imported through electronic medical records in most cases [28, 48]. Chan et al. noted that the implementation of KMC was promoted when medical units improved their electronic medical records to allow nurses to record the onset and duration of KMC [28]. Therefore, the Ministry of Health and government agencies should formulate practical KMC implementation guidelines based on local conditions, and medical units should also formulate and standardize KMC implementation guidelines and programs to promote the implementation of KMC.

Lack of proper leadership, insufficient professionalism of personnel, and insufficient training were also obstacles to KMC implementation. A study on the introduction of KMC in Indonesian hospitals found that government support, hospital management, staff acceptance, and training were identified as key facilitators of KMC implementation [47]. In some regions, KMC-specific training programs were provisioned for medical staff by the government [49]. However, the number of staff participating in the training is very limited due to the long distance between the training site and the medical unit and the shortage of personnel in the hospitals, although many medical personnel were willing to participate in the training [42, 50]. In other words, although policymakers and decision-makers tried to provide assistance and intervention programs for healthcare workers, they did not anticipate these barriers to attendance. Of course, the support from hospital administrators and leadership could provide more space and human resources to provision KMC, optimize or update the staffing configuration of neonatal care nurses, strengthen the professionalization of neonatal care by healthcare workers, and improve healthcare staff’s attitudes towards and perceptions of KMC [43, 51].

The attitudes of the health caregivers towards KMC were also a factor influencing the adoption of KMC for parents. If there were staff in the hospital who were familiar with KMC and willing to educate parents on KMC knowledge, it would help parents of preterm infants to acquire KMC-related knowledge, which would promote KMC preferences and the early initiation of KMC [52, 53]. Correspondingly, insufficient awareness of KMC and infant health among parents/family members was a barrier to the practice of KMC [22]. Despite the generally low awareness of KMC, the reviews reported that it was relatively easier to train mothers on KMC practices and that they were more adherent to KMC practices after understanding and accepting KMC [54]. Perceived, observed, and experienced effects of KMC could provide comfort and satisfaction to the parents of preterm infants, which promotes KMC use, whereas KMC is inhibited if parents and/or preterm infants experience KMC-related discomfort.

Lack of assistance is a barrier to KMC practice, whereas support from family, friends, and other mothers is a facilitator to the implementation of KMC. There were many different forms of support. For example, family members took turns embracing the preterm infants to free the mother from this practice [55, 56]. Evidence from the literature has suggested that emotional support, as well as support and help with household chores, is also a facilitator for mothers [57, 58]. Kangaroo nursing can be implemented not only by mothers but also by fathers, grandfathers, grandmothers, and other family members of preterm infants [43, 59], and if family members do not understand this point, preterm infants might lose the opportunity to receive kangaroo care [60]. Therefore, different educational approaches should be adopted to educate families of preterm infants about their roles in KMC, with additional health promotions and activities targeting grandparents and other family members about the benefits of KMC and the significance of supporting mothers, which may increase the number of people receiving KMC.

However, KMC is not suitable for all situations. In some clinical scenarios where mothers of preterm infants have special health conditions, it could be very challenging to train mothers and facilitate KMC implementation. These challenges include the infant being too difficult to embrace, the infant being too heavy, and the mother experiencing chest or back discomfort or pain/fatigue [38]. The reviews showed that mothers’ medical conditions, including postepisiotomy pain repair [61], postcesarean recovery [62], postpartum depression and general maternal illness [48], were another challenge for KMC practice. Additionally, mothers may mentally struggle with KMC practices, including positioning problems (difficulty sleeping on the chest with infants), breast milk expression, and other breastfeeding-related issues [57, 63]. In this case, family support and father involvement make a great difference [64]. Postpartum depression is a barrier to the implementation of KMC, but interestingly, mothers who practised KMC experienced reduced symptoms of postpartum depression [65, 66].

Inviting parents to the NICU to perform KMC could result in extra costs. Studies performed in low-income countries have shown that commuting between home and KMC wards was a barrier to the implementation of KMC, and fees for mothers and babies staying in KMC wards were also considered a barrier [39, 67]. Studies have shown that higher economic status is more conducive to the implementation of KMC [40, 43]. Therefore, accessing financial resources from hospital administration and/or parental health insurance to facilitate KMC would be a necessary part of KMC expansion. Meanwhile, it is necessary to consider how to reduce hospital charges or provide certain transportation subsidies for families with infants whose hospitalization time exceeds the average length of stay. Limited visiting time in the NICU is another obstacle to the implementation of KMC, especially in the case of closed management such as the NICU in China. Extending the visit time could increase the adoption of KMC to some extent [68, 69].

Different cultures, religions, and traditional beliefs in different countries influence perceptions of preterm infants and KMC. In many countries, carrying infants on the chest rather than on the back is considered inappropriate [41], and some cultures believe that skin-to-skin contact between an infant and his or her caregiver is not appropriate [27]. Understanding these culturally specific barriers, it is of great importance to adapt KMC promotion programmes to the needs of the population. In some countries, mothers are ridiculed for giving birth to preterm infants, which results in stigma [55, 70]. Studies have reported that stigma about preterm infants creates anxiety and guilt in mothers, causing them to abandon their infants, which is a factor hindering the implementation of KMC [27, 38]. Muddu et al. [71] found that fondness was an enabler for parents to accept their preterm infants and utilize KMC to support the improvement of their preterm infants’ health. Cultural barriers also encompass the practice of postpartum confinement and traditional resistance to confinement from grandparents and community members. Most mothers are advised to stay home after delivery in China and India [72, 73], which has potential health benefits for mothers and newborns, but it also causes mothers and families to be hesitant to adopt KMC.

Traditional gender role factors were identified as barriers to male participation in neonatal care. KMC was regarded as a breach of social duty or responsibility by mothers in some countries where it is believed that mothers should take care of the family, and when mothers comply with this social duty and gender responsibility, the implementation of KMC becomes a challenge [74]; meanwhile, fathers are not encouraged to participate in KMC implementation in such cultures. Therefore, it is of great significance to develop interventions on how to encourage fathers to participate in KMC and reduce the stigma surrounding this infant care strategy [75]. As Dumbaugh et al. [76] pointed out, the inclusion of males in neonatal care must be done in a way that empowers women. Fathers who are successfully involved in KMC might become peer mentors or examples for others to address the problem of fathers’ reluctance to participate in neonatal care. The name of the intervention, “kangaroo mother care”, could also be modified, e.g., to “kangaroo care”, so that it does not directly imply that the practice is performed only by mothers.

## Limitations

The findings in this manuscript are subject to some limitations. First, due to resource constraints, we only searched for English and Chinese reviews, and there was a possibility of missing some relevant studies. Another limitation of the umbrella review approach was that it could only report on what researchers have investigated and published [32]. For example, some factors might be highly influential, but if they were not adequately investigated in the included studies, they might be reported as less important, or they might not even be included in the review. To mitigate this issue, other key literature not identified in this review was actively referenced. Finally, a potential limitation to the umbrella review approach could be the risk that bias is transmitted upwards from primary studies to the reviews and then to the umbrella review.

## Recommendations for future research

KMC implementation issues are likely to differ among different regions, so there remains a need for further research into sustainable development mechanisms in varied settings to promote the adoption of KMC. The generalizability of the findings worldwide and their translation into practice is uncertain. Most of the studies focused at the facility level, such as the NICU, which highlights the lack of community-level studies. Therefore, further research is needed to explore the factors influencing KMC implementation at home and in the community. Male involvement was identified as a facilitator to KMC implementation, but there was no study discussing hindrance factors of father involvement in care specifically. Therefore, further research is also needed to explore the hindrance and/or facilitating factors of male involvement in KMC care from the perspective of fathers. In addition, further research is also needed to test models for addressing barriers and supporting facilitators to promote and implement context-specific health system changes for greater uptake of KMC.

## Conclusions

KMC is a complicated intervention that encounters unique barriers and facilitators in different aspects of healthcare systems. Our umbrella review prioritizes the main factors influencing KMC implementation and highlights some key areas that implementers and implementation researchers may need to focus on. KMC should be implemented more systematically and continuously to strengthen and expand its adoption.

The parents of preterm infants and other family members, the medical unit, and the medical staff contribute to a dynamic whole as a triangle, that are closely linked with one another. Support from facility management and leadership and well-trained medical staff are of great significance to the successful integration of KMC into daily medical practice, while the parents of preterm infants and other family members should be educated and encouraged to adopt KMC practice. Effectively integrating KMC into current health systems by addressing barriers and building trust will greatly improve neonatal survival rates.

## Supplementary Information

**Additional file 1.** PRISMA 2020 Checklist.

**Additional file 2.** Search strategies for English and Chinese databases.

**Additional file 3.** 1. Articles presenting barriers to implementing KMC. 2. Articles presenting facilitators to implementing KMC.

**Additional file 4.** Result of the quality appraisal of included studies.

**Additional file 5.** Risk of Bias analysis using ROBIS tool.

## Data Availability

The datasets analyzed during the current study are available from the corresponding author on reasonable request. All data were extracted from published systematic reviews and meta-analyses.
