# Supplementary material for: What influences the implementation of kangaroo mother care? An umbrella review
Source: BMC Pregnancy Childbirth. 2022 Nov 18;22:851. doi: 10.1186/s12884-022-05163-3 (PMC9675107; doi:10.1186/s12884-022-05163-3)
Supplement: Supplementary file 2 — Additional file 2. Search strategies for English and Chinese databases. [file 12884_2022_5163_MOESM2_ESM.docx]

**Additional file2: Search strategies for English and Chinese databases.**

| **Database** | **Query** | **Items found** |
| --- | --- | --- |
| PubMed | #1 "Intensive Care, Neonatal"[Mesh] OR "Intensive Care Units, Neonatal"[Mesh] OR "Premature Birth"[Mesh] OR "Infant, Newborn"[Mesh] OR "Infant, Premature"[Mesh] OR "Infant, Extremely Premature"[Mesh] OR "Infant, Low Birth Weight"[Mesh] OR prematur*[Title/Abstract] OR preterm*[Title/Abstract] OR premie*[Title/Abstract] OR neonat*[Title/Abstract] OR infant*[Title/Abstract] OR newborn*[Title/Abstract] OR "low birth weight"[Title/Abstract] OR LBW[Title/Abstract] OR NICU[Title/Abstract] | 1215787 |
|  | #2 "Kangaroo-Mother Care Method"[Mesh] OR "kangaroo mother care"[Title/Abstract] OR "kangaroo mother method"[Title/Abstract] OR "kangaroo care"[Title/Abstract] OR "kangaroo attachment"[Title/Abstract] OR "kangaroo contact"[Title/Abstract] OR KMC[Title/Abstract] OR KC[Title/Abstract] OR "skin-to-skin care"[Title/Abstract] OR "skin-to-skin contact"[Title/Abstract] OR SSC[Title/Abstract] OR "mother-infant contact"[Title/Abstract] | 26629 |
|  | #3 "Systematic Reviews as Topic"[Mesh] OR "Systematic Review"[Publication Type] OR "Meta-Analysis as Topic"[Mesh] OR "Meta-Analysis"[Publication Type] OR "systematic review"[Title/Abstract] OR "meta-analys*"[Title/Abstract] | 400303 |
|  | #4 #1 AND #2 AND #3 | 154 |
| Embase | #1 'newborn intensive care'/exp OR 'neonatal intensive care unit'/exp OR 'prematurity'/exp OR 'newborn'/exp OR 'low birth weight'/exp OR 'prematur*':ti,ab,kw OR 'preterm*':ti,ab,kw OR 'premie*':ti,ab,kw OR 'neonat*':ti,ab,kw OR 'infant*':ti,ab,kw OR 'newborn*':ti,ab,kw OR 'low birth weight':ti,ab,kw OR 'lbw':ti,ab,kw OR 'nicu':ti,ab,kw | 1461179 |
|  | #2 'kangaroo care'/exp OR 'kangaroo mother care':ti,ab,kw OR 'kangaroo mother method':ti,ab,kw OR 'kangaroo care':ti,ab,kw OR 'kangaroo attachment':ti,ab,kw OR 'kangaroo contact':ti,ab,kw OR 'kmc':ti,ab,kw OR 'kc':ti,ab,kw OR 'skin-to-skin care':ti,ab,kw OR 'skin-to-skin contact':ti,ab,kw OR 'ssc':ti,ab,kw OR 'mother-infant contact':ti,ab,kw | 39972 |
|  | #3 'systematic review'/exp OR 'systematic review (topic)'/exp OR 'meta analysis'/exp OR 'meta analysis (topic)'/exp OR 'systematic review':ti,ab,kw OR 'meta-analys*':ti,ab,kw | 617260 |
|  | #4 #1 AND #2 AND #3 | 211 |
| CINAHL | #1 (MH "Intensive Care, Neonatal+") OR (MH "Intensive Care Units, Neonatal") OR (MH "Infant, Premature") OR (MH "Childbirth, Premature") OR (MH "Infant, Low Birth Weight+") OR (MH "Infant, Very Low Birth Weight") OR (MH "Infant, Newborn+") OR TI prematur* OR TI preterm* OR TI premie* OR TI neonat* OR TI infant* OR TI newborn* OR TI "low birth weight" OR TI LBW OR TI NICU OR AB prematur* OR AB preterm* OR AB premie* OR AB neonat* OR AB infant* OR AB newborn* OR AB "low birth weight" OR AB LBW OR AB NICU | 293151 |
|  | #2 (MH "Kangaroo Care") OR TI "kangaroo mother care" OR TI "kangaroo mother method" OR TI "kangaroo care" OR TI "'kangaroo attachment" OR TI "kangaroo contact" OR TI KMC OR TI KC OR TI "skin-to-skin care" OR TI "skin-to-skin contact" OR TI SSC OR TI "mother-infant contact" OR AB "kangaroo mother care" OR AB "kangaroo mother method" OR AB "kangaroo care" OR AB "'kangaroo attachment" OR AB "kangaroo contact" OR AB KMC OR AB KC OR AB "skin-to-skin care" OR AB "skin-to-skin contact" OR AB SSC OR AB "mother-infant contact" | 5984 |
|  | #3 (MH "Systematic Review") OR (MH "Cochrane Library") OR (MH "Meta Analysis") OR (MH "Meta Synthesis") OR TI "systematic review" OR TI "meta-analys*" OR AB "systematic review" OR AB "meta-analys*" | 198060 |
|  | #4 #1 AND #2 AND #3 | 134 |
| Cochrane | #1 MeSH descriptor: [Intensive Care, Neonatal] explode all trees OR MeSH descriptor: [Intensive Care Units, Neonatal] explode all trees OR MeSH descriptor: [Premature Birth] explode all trees OR MeSH descriptor: [Infant, Newborn] explode all trees OR MeSH descriptor: [Infant, Premature] explode all trees OR MeSH descriptor: [Infant, Extremely Premature] explode all trees OR MeSH descriptor: [Infant, Low Birth Weight] explode all trees OR (prematur*):ti,ab,kw OR (preterm*):ti,ab,kw OR (premie*):ti,ab,kw OR (neonat*):ti,ab,kw OR (infant*):ti,ab,kw OR (newborn*):ti,ab,kw OR ("low birth weight"):ti,ab,kw OR (LBW):ti,ab,kw OR (NICU):ti,ab,kw | 95732 |
|  | #2 MeSH descriptor: [Kangaroo-Mother Care Method] explode all trees OR (kangaroo mother care):ti,ab,kw OR (kangaroo mother method):ti,ab,kw OR (kangaroo care):ti,ab,kw OR (kangaroo attachment):ti,ab,kw OR (kangaroo contact):ti,ab,kw OR (KMC):ti,ab,kw OR (KC):ti,ab,kw OR (skin-to-skin care):ti,ab,kw OR (skin-to-skin contact):ti,ab,kw OR (SSC):ti,ab,kw OR (mother-infant contact):ti,ab,kw | 2562 |
|  | #3 MeSH descriptor: [Systematic Reviews as Topic] explode all trees OR MeSH descriptor: [Meta-Analysis as Topic] explode all trees OR (systematic review):ti,ab,kw OR (meta-analys*):ti,ab,kw | 28358 |
|  | #4 #1 AND #2 AND #3 | 29 |
| CNKI | #1 TKA=('早产儿' + '新生儿' + '低出生体重儿')*('袋鼠护理' + '袋鼠式护理' + '皮肤接触')*('系统评价' + '系统综述' + '荟萃分析' + 'Meta分析') | 13 |
| WAN FAN | #1 主题:("早产儿" or "新生儿" or "低出生体重儿") and 主题:("袋鼠护理" or "袋鼠式护理" or "皮肤接触") and 主题:("系统评价" or "系统综述" or "荟萃分析" or "Meta分析") | 19 |
| SinoMed | #1 ("早产儿"[常用字段:智能] OR "新生儿"[常用字段:智能] OR "低出生体重儿"[常用字段:智能]) AND ("袋鼠护理"[常用字段:智能] OR "袋鼠式护理"[常用字段:智能] OR "皮肤接触"[常用字段:智能]) AND ("系统评价"[常用字段:智能] OR "系统综述"[常用字段:智能] OR "荟萃分析"[常用字段:智能] OR "Meta分析"[常用字段:智能]) | 11 |
