# Supplementary material for: What influences the implementation of kangaroo mother care? An umbrella review
Source: BMC Pregnancy Childbirth. 2022 Nov 18;22:851. doi: 10.1186/s12884-022-05163-3 (PMC9675107; doi:10.1186/s12884-022-05163-3)
Supplement: Supplementary file 3 — Additional file 3. 1. Articles presenting barriers to implementing KMC. 2. Articles presenting facilitators to implementing KMC. [file 12884_2022_5163_MOESM3_ESM.docx]

**Additional file3-1: Articles presenting barriers to implementing KMC.**

| **Factors** | **Studies identified** | | | | | |
| --- | --- | --- | --- | --- | --- | --- |
|  | Smith et al. [27] | Chan et al.  [28] | Seidman et al.  [38] | Kinshella et al.  [29] | Mathias et al.  [39] | Chan et al.  [40] |
| **1. Environmental Factors** |  |  |  |  |  |  |
| **1.1 Facility conditions** |  |  |  |  |  |  |
| Lack of privacy | √ | √ |  |  | √ | √ |
| Space limitations induced discharge within hours |  | √ |  |  |  |  |
| Crowding and insufficient space in the NICU |  | √ |  |  |  | √ |
| Issues with facility environment/resources |  |  | √ |  |  |  |
| Temperature |  |  | √ |  |  |  |
| Issues with clothing/infants’ medical devices |  |  | √ |  |  |  |
| Logistical issues related to implementing new practice |  |  | √ |  |  |  |
| Inadequate facilities and supplies |  |  |  | √ |  |  |
| **1.2 Resources and Materials** |  |  |  |  |  |  |
| Lack of necessary resources | √ |  | √ |  |  |  |
| KMC protocols perceived as  inflexible |  | √ |  |  |  |  |
| Few NICUs had written KMC protocols |  | √ |  |  |  |  |
| No checklist for KMC admission procedures |  | √ |  |  |  |  |
| Staff need to bargain with managers to increase and maintain resources for newborn care |  | √ |  |  |  |  |
| KMC was not budgeted for, and resources were mismanaged |  | √ |  |  |  |  |
| No record of SSC |  | √ |  |  |  |  |
| Difficulty adapting/teaching electronic medical records for KMC |  | √ |  |  |  |  |
| Lack of KMC guidelines |  |  | √ | √ |  |  |
| Record-keeping challenges for HCWs |  |  | √ |  |  |  |
| Facilities did not provide food for mothers |  |  |  |  |  | √ |
| Poor management of resources donated to the hospital |  |  |  |  |  | √ |
| Lack of use of data to document skin-to-skin contact practised on electronic medical record |  |  |  |  |  | √ |
| **1.3 Healthcare system** |  |  |  |  |  |  |
| Inconsistent application of KMC within facilities and among HCWs |  | √ |  |  |  |  |
| Visitation policies were difficult due to strained communication between parents and staff |  | √ |  |  |  |  |
| Staff not trained in preterm care |  | √ |  |  |  |  |
| Follow-up and discharge procedures not well structured |  | √ |  |  | √ |  |
| KMC training not part of a broader healthcare training curriculum |  | √ |  |  |  |  |
| Poor training lead to conflicting knowledge on time and duration of SSC |  | √ |  |  |  |  |
| Implementing continuous KMC was difficult. Many facilities reported performing continuous KMC, but few actually practiced it |  | √ |  |  |  |  |
| Receiving visitors |  |  | √ |  |  |  |
| Unsupportive staffing policies |  |  |  | √ |  |  |
| Poor supportive supervision and record-keeping |  |  |  | √ |  |  |
| Inadequate/inconsistent training |  |  |  |  | √ | √ |
| Inconsistent knowledge and application of kangaroo mother care |  |  |  |  |  | √ |
| Only low birthweight infants received kangaroo mother care in some locations |  |  |  |  |  | √ |
| Visitation policies sometimes prevented mothers from performing skin-to-skin contact continuously |  |  |  |  |  | √ |
| **2. Professional Factors** |  |  |  |  |  |  |
| **2.1 Professional Perception** |  |  |  |  |  |  |
| Nurses believe KMC based on perception and not scientific fact |  | √ |  |  |  |  |
| Concerns on the stability of the infant |  | √ |  |  |  |  |
| Some HCWs considered parents and visitors as a barrier |  | √ |  |  |  |  |
| Did not believe KMC was safe for LBW newborns |  | √ |  |  |  |  |
| Concerns about other medical conditions/care |  |  | √ |  |  |  |
| General lack of buy-in/belief in efficacy |  |  | √ |  |  |  |
| Belief that KMC causes extra work |  |  | √ |  |  |  |
| Low awareness/misinformation about practice |  |  | √ |  |  |  |
| Concerns about parents’ ability to practice |  |  | √ |  |  |  |
| Health worker attitudes and non-acceptance |  |  |  | √ |  |  |
| KMC perceived not safe and causes infection and neck deformity |  |  |  |  | √ |  |
| Medical stabilisation of LBWI perceived as restriction to KMC initiation |  |  |  |  | √ |  |
| Nurses fail to have strong belief in importance of kangaroo mother care |  |  |  |  |  | √ |
| Parents could serve as a hindrance to health-care worker |  |  |  |  |  | √ |
| Nurses did not feel kangaroo mother care appropriate for infants who they felt were too small/young/ill |  |  |  |  |  | √ |
| Disagreement over clinical stability |  |  |  |  |  | √ |
| Staff found visitors get in the way |  |  |  |  |  | √ |
| **2.2 Professional Characteristics** |  |  |  |  |  |  |
| Unsupportive, loud, uncaring | √ |  |  |  |  |  |
| Limited communication between HCWs |  | √ |  |  |  |  |
| Level of experience |  |  | √ |  |  |  |
| Lack of change mindset |  |  | √ |  |  |  |
| Inadequate knowledge |  |  |  | √ |  |  |
| Staff resisted changing protocols |  |  |  |  |  | √ |
| Nurses not given feedback on kangaroo mother care data collected |  |  |  |  |  | √ |
| **2.3 Professional Management** |  |  |  |  |  |  |
| Management reluctance to allocate space for SSC |  | √ |  |  |  |  |
| High staff and leadership turnover |  | √ |  |  |  | √ |
| Lack of leadership and support from management |  | √ |  |  |  |  |
| Newborn care was not a priority in the health system |  | √ |  | √ |  |  |
| Lack of buy-in from opinion leaders |  |  | √ |  |  |  |
| Handoff issues with other nurses |  |  | √ |  |  |  |
| Need for high-touch support from staff |  |  | √ |  |  |  |
| Lack of local leadership |  |  |  | √ |  |  |
| Leadership lack of buy-in led to lack of adequate resources |  |  |  |  |  | √ |
| Management did not prioritize kangaroo mother care |  |  |  |  |  | √ |
| **3. Parents/Family Factors** |  |  |  |  |  |  |
| **3.1 Perception and Motivation** |  |  |  |  |  |  |
| Were unaware of the benefits of KMC | √ |  |  |  |  |  |
| Were expected to perform KMC with little or no instruction | √ |  |  |  |  |  |
| Could not see newborn during KMC | √ |  |  |  |  |  |
| Did not feel a bond with the infant | √ |  |  |  |  |  |
| Perceived newborn did not enjoy KMC | √ |  |  |  |  |  |
| Mothers lonely and depressed in KMC ward | √ |  |  |  |  | √ |
| Negative impressions of staff attitudes or interactions |  |  | √ |  |  |  |
| Fear/anxiety of hurting the infant |  |  | √ |  |  |  |
| Lack of awareness of KMC/infant health |  |  | √ | √ | √ |  |
| Discomfort/unease with the situation |  |  | √ |  |  |  |
| Isolation effect |  |  | √ |  |  |  |
| Fears and discomforts with KMC practice |  |  |  | √ |  |  |
| Felt less of women for having LBWIs |  |  |  |  | √ |  |
| Maternal attitude towards KMC |  |  |  |  | √ |  |
| Experienced and perceived discomforts to the parent and/or LBWI associated with KMC |  |  |  |  | √ |  |
| PLBWI ridiculed by the family and community |  |  |  |  | √ |  |
| **3.2 Parenting Capacity** |  |  |  |  |  |  |
| Pain/fatigue | √ |  | √ |  |  | √ |
| Mother’s medical issues/post-partum depression | √ |  | √ | √ |  |  |
| Pain hindered KMC, particularly after a C-section | √ |  |  |  |  |  |
| Discomfort sleeping upright | √ |  |  |  |  |  |
| Positioning issues (including sleeping) |  |  | √ |  |  |  |
| Demographics of mother or infant |  |  | √ |  |  |  |
| Breastmilk expression and others BF-related issues |  |  | √ |  |  |  |
| Low self-esteem and lack of confidence |  |  |  |  | √ |  |
| Lack of knowledge on KMC |  |  |  |  | √ |  |
| **3.3 Support and empowerment** |  |  |  |  |  |  |
| HCWs Did not respect family privacy | √ |  |  |  |  | √ |
| Mothers-in-law and grandmothers did not approve | √ |  |  |  |  |  |
| Bad attitudes and peer pressure negatively influenced desire to perform KMC | √ |  |  |  |  |  |
| Lack of help with KMC practice and other obligations |  |  | √ |  |  |  |
| General lack of buy-in/low perceived value |  |  | √ |  |  |  |
| Disapproval from community |  |  | √ |  |  |  |
| Disempowerment in decision-making |  |  | √ | √ | √ |  |
| Family attitudes |  |  |  | √ |  |  |
| Poor support or negative interactions with medical staff |  |  |  | √ |  |  |
| Lack of family support |  |  |  |  | √ |  |
| **4. Access Factors** |  |  |  |  |  |  |
| **4.1 Time/Workload** |  |  |  |  |  |  |
| Caregivers unable to devote time | √ |  |  |  |  | √ |
| Time needed to commute from home to hospital was too much | √ |  |  |  |  |  |
| Training mothers to do SSC would take additional time out of health workers’ schedules, increase their workload, and reduce time with other critical patients |  | √ |  |  |  |  |
| Shortage of staff nurses limited parental access and shortened visitation time |  | √ |  |  | √ | √ |
| The shorter the visitation period was, the more of an interference staff thought parents were |  | √ |  |  |  |  |
| Actual increased workload/staff shortages |  |  | √ | √ |  | √ |
| Stresses related to extended hospitalization |  |  |  | √ |  |  |
| The season of the year |  |  |  |  | √ |  |
| Long hospitalisation stay |  |  |  |  | √ |  |
| KMC consumes time for house chores |  |  |  |  | √ |  |
| Takes away time from other patients |  |  |  |  |  | √ |
| Health-care workers has difficulty finding time for training |  |  |  |  |  | √ |
| **4.2 Location** |  |  |  |  |  |  |
| Other responsibilities at home or work interfered | √ |  |  |  |  |  |
| Home delivery: late/delayed KMC initiation |  |  |  |  | √ |  |
| **4.3 Financing** |  |  |  |  |  |  |
| Cost associated with travel, food, lodging, parking, clinical fees | √ |  |  |  |  | √ |
| Lack of transport and distance to facility | √ |  |  |  |  | √ |
| Difficulty accessing facility |  |  | √ |  |  |  |
| Lack of money for transportation, beds and kangaroo mother care wrappers |  |  |  |  |  | √ |
| **5. Cultural Factors** |  |  |  |  |  |  |
| **5.1 Traditional newborn care** |  |  |  |  |  |  |
| Infants traditionally carried on back, thus carrying on the front seemed odd | √ |  |  |  |  |  |
| Bathing practices interfered | √ |  |  |  |  |  |
| If breast feeding not pursued KMC less likely to continue | √ |  |  |  |  |  |
| Bathing practices and wrapping infants soon after birth delayed SSC |  | √ |  |  |  |  |
| Type of wrap: traditional chitenje |  |  |  |  | √ |  |
| Traditional bathing, carrying and breastfeeding practices did not always align with kangaroo mother care guidelines |  |  |  |  |  | √ |
| Traditional protocols interfered (bathing, carrying) |  |  |  |  |  | √ |
| **5.2 Traditional mindset** |  |  |  |  |  |  |
| Mothers reported shame of having a preterm infant | √ |  |  |  |  |  |
| Fear, guilt doing KMC publically | √ |  |  |  |  | √ |
| Considered unclean where diapers not used | √ |  |  |  |  |  |
| Stigma | √ |  | √ |  |  |  |
| In warm climates staff did not believe hat and socks were necessary |  | √ |  |  |  |  |
| Country or culture-specific beliefs, practices, or policies |  |  | √ | √ |  |  |
| Cultural association of infants skin rash to mother–infant skin contact |  |  |  |  | √ |  |
| KMC hinders social obligations |  |  |  |  | √ |  |
| Cultural/traditional belief of waiting for the umbilical cord to fall off before KMC started |  |  |  |  | √ |  |
| KMC considered as taboo |  |  |  |  | √ |  |
| Stigma, shame, kangaroo mother care felt forced |  |  |  |  |  | √ |
| **5.3 Gender Roles** |  |  |  |  |  |  |
| Felt KMC was role of mother | √ |  |  |  |  |  |
| Mothers did not want father to perform KMC | √ |  |  |  |  |  |
| Issues related to gender roles | √ |  | √ |  |  |  |
| Fathers lack of opportunity to practice |  |  | √ |  |  |  |
| The males not allowed in the KMC room |  |  |  |  | √ |  |
| Lack of male involvement |  |  |  |  | √ |  |
| Nurse excluding father from infant care was a cultural norm |  |  |  |  |  | √ |

**Additional file3-2: Articles presenting facilitators to implementing KMC.**

| **Factors** | **Studies identified** | | | | | |
| --- | --- | --- | --- | --- | --- | --- |
|  | Smith  et al.  [27] | Chan et al.  [28] | Seidman et al.  [38] | Kinshella et al.  [29] | Mathias et al.  [39] | Chan et al.  [40] |
| **1. Environmental Factors** |  |  |  |  |  |  |
| **1.1 Facility conditions** |  |  |  |  |  |  |
| Private, quiet spaces for KMC | √ |  |  |  |  |  |
| KMC ward |  | √ |  |  |  |  |
| Access to private space/ privacy screens |  | √ |  |  |  | √ |
| Relaxed atmosphere with dim lighting |  | √ |  |  |  |  |
| Positive elements of the facility |  |  | √ |  |  |  |
| Sufficient space and supplies |  |  |  | √ |  |  |
| Temperature stability |  |  |  |  |  | √ |
| Quiet atmosphere within facilities allows mothers to rest |  |  |  |  |  | √ |
| **1.2 Resources and materials** |  |  |  |  |  |  |
| Posters of KMC in the facility |  | √ |  |  |  |  |
| Management mobilization of resources |  | √ |  |  |  |  |
| Use of technology |  | √ |  |  |  |  |
| Use of KMC guidelines or protocols |  | √ |  | √ |  |  |
| Use of KMC expert clients |  |  |  |  | √ |  |
| Displayed KMC pictures/posters |  |  |  |  | √ |  |
| KMC pictorial presentations and photographs |  |  |  |  | √ |  |
| Recreation activities |  |  |  |  | √ |  |
| Access to structural resources |  |  |  |  |  | √ |
| Breast milk banks provide milk and can be an educational tool among mothers |  |  |  |  |  | √ |
| Reporting and data |  |  |  |  |  | √ |
| Collection of data |  |  |  |  |  | √ |
| Site assessment tools |  |  |  |  |  | √ |
| **1.3 Healthcare system** |  |  |  |  |  |  |
| Expanding training to other healthcare personnel besides nurses |  | √ |  |  |  |  |
| Include KMC in health facility statistics |  | √ |  |  |  |  |
| KMC policies |  |  |  | √ |  |  |
| Supportive staffing policies |  |  |  | √ |  |  |
| Supportive Supervision and dedicated registers |  |  |  | √ |  |  |
| Integrating into maternal health services |  |  |  | √ |  |  |
| Follow-up at the facility-based KMC |  |  |  |  | √ |  |
| Educating the key influential community members |  |  |  |  | √ |  |
| Ongoing KMC education at facility-based care |  |  |  |  | √ |  |
| Virtual communication and training |  |  |  |  |  | √ |
| Integration of kangaroo mother care into health-care curriculum |  |  |  |  |  | √ |
| Use of performance standards and quality improvement measures |  |  |  |  |  | √ |
| **2. Professional Factors** |  |  |  |  |  |  |
| **2.1 Professional Perception** |  |  |  |  |  |  |
| Nurses were more likely to perform KMC if they believed it worked |  | √ |  |  |  |  |
| Shorter newborns crying times in response to pain with KMC compared with incubator care |  | √ |  |  |  |  |
| Adequate training and understanding of KMC benefits |  |  |  | √ |  |  |
| Nurses more likely to use kangaroo mother care after seeing positive effects |  |  |  |  |  | √ |
| **2.2 Professional Characteristics** |  |  |  |  |  |  |
| Experience with KMC |  | √ |  |  |  | √ |
| Nurses with 5 or more years of experience more likely to implement KMC |  | √ |  |  |  |  |
| Staff acceptability and enthusiasm |  |  |  | √ |  |  |
| Nurses’ willingness to educate PLBWIs |  |  |  |  | √ |  |
| Good communication |  |  |  |  |  | √ |
| **2.3 Professional Management** |  |  |  |  |  |  |
| Nurse involvement in care related decision making |  | √ |  |  |  |  |
| Multiple health worker support facilitated SSC - nutrition workers, CHWs and clinical workers |  | √ |  |  |  |  |
| Practicing securing catheters lowered nurses’ concerns |  | √ |  |  |  |  |
| Local leadership support |  |  |  | √ |  |  |
| Mentorship and opportunities to share knowledge |  |  |  | √ |  |  |
| Availability of skilled KMC health workers |  |  |  |  | √ |  |
| Availability of KMC providers, resources, Nurses’ empathy |  |  |  |  | √ |  |
| KMC support groups facilitated KMC utilisation |  |  |  |  | √ |  |
| Leadership and management support |  |  |  |  |  | √ |
| Management promotion of kangaroo mother care |  |  |  |  |  | √ |
| Use of committees to advocate for kangaroo mother care |  |  |  |  |  | √ |
| **3. Parents/Family Factors** |  |  |  |  |  |  |
| **3.1 Perception and Motivation** |  |  |  |  |  |  |
| Newborns slept longer, less anxious, happier, more willing to feed | √ |  |  |  |  |  |
| KMC was calming, relaxing, comforting, natural, instinctive, secure, logical, healing | √ |  |  |  |  |  |
| Created a family bond, inspired caregiver confidence | √ |  |  |  |  |  |
| Sped emotional and physical recovery of mother | √ |  |  |  |  |  |
| Made caregivers feel useful | √ |  |  |  |  |  |
| Mother-infant attachment |  |  | √ |  | √ |  |
| Feelings of confidence/ empowerment |  |  | √ | √ |  |  |
| Ease of practice/preference over traditional care |  |  | √ |  |  |  |
| Understanding of efficacy/ benefits |  |  | √ | √ |  |  |
| Belief that infant enjoys practice |  |  | √ |  |  |  |
| Ease of teaching/training mothers |  |  | √ |  |  |  |
| Awareness of KMC before birth |  |  | √ |  |  |  |
| Early discharge as motivator |  |  | √ |  |  |  |
| Positive attitudes toward PT survival |  |  | √ |  |  |  |
| KMC preference |  |  |  |  | √ | √ |
| Affection towards the LBWIs |  |  |  |  | √ |  |
| Perceived and experienced KMC benefits |  |  |  |  | √ |  |
| KMC awareness |  |  |  |  | √ |  |
| Calming, natural, instinctive, healing for parents and infant |  |  |  |  |  | √ |
| **3.2 Parenting Capacity** |  |  |  |  |  |  |
| KMC helped mother’s recover from post-partum depression | √ |  |  |  |  |  |
| KMC helped to relieve stress and promote emotional well-being | √ |  |  |  |  |  |
| Ability to stay with infant |  |  | √ |  |  |  |
| Maternal confidence/will to practice KMC |  |  |  |  | √ |  |
| KMC knowledge |  |  |  |  | √ |  |
| Health seeking behaviour |  |  |  |  | √ |  |
| Managing postpartum pains |  |  |  |  | √ |  |
| Helped mothers recover emotionally |  |  |  |  |  | √ |
| **3.3 Support and empowerment** |  |  |  |  |  |  |
| Best results with continuous training and support from HCWs | √ |  |  |  |  |  |
| Grandmothers, sisters, others helping with chores increased uptake and duration of KMC | √ |  |  |  |  |  |
| Paternal support crucial to success of KMC, they alleviate workload, support, encourage, increase mother’s confidence | √ |  |  |  |  |  |
| Family more likely to understand and respond well if mother explained KMC | √ |  |  |  |  |  |
| Companions for mothers promoted KMC |  | √ |  |  |  |  |
| Support from family, friends, and other mothers |  | √ |  |  |  |  |
| Support from staff or community health worker (CHW) |  | √ |  |  |  |  |
| Access to staff and training on KMC |  |  | √ |  |  |  |
| Improved family interactions |  |  | √ |  |  |  |
| Support from government/ employers for parents |  |  | √ |  |  |  |
| Family support and visits |  |  |  | √ |  |  |
| Receiving support from medical staff |  |  |  | √ |  |  |
| Peer support from other mothers |  |  |  | √ |  |  |
| Incorporating mothers in decision making on LBWIs’ care |  |  |  |  | √ |  |
| Good nurse-mother relationship |  |  |  |  | √ |  |
| Return demonstration |  |  |  |  | √ |  |
| KMC support and encouragementh |  |  |  |  | √ |  |
| Woman empowerment: decision-making |  |  |  |  | √ |  |
| Family and community support with KMC practice |  |  |  |  | √ |  |
| Prior identified support system |  |  |  |  | √ |  |
| Father, health-care worker, family and community support for mothers and fathers was crucial to success of kangaroo mother care |  |  |  |  |  | √ |
| Role of parents and other health-care workers |  |  |  |  |  | √ |
| Staffing support |  |  |  |  |  | √ |
| **4. Access Factors** |  |  |  |  |  |  |
| **4.1 Time/Workload** |  |  |  |  |  |  |
| Unlimited visitation hours at health facility | √ | √ |  |  |  | √ |
| Some nurses reported that KMC did not increase the amount of time they spent on each patient |  | √ |  |  |  |  |
| Early KMC initiation |  |  |  |  | √ |  |
| Kangaroo mother care did not increase workload |  |  |  |  |  | √ |
| **4.2 Location** |  |  |  |  |  |  |
| Parents preferred to practice KMC at home than at the facility to at tend to other responsibilities | √ |  |  |  |  |  |
| Hospital delivery: prompt KMC uptake |  |  |  |  | √ |  |
| Kangaroo mother care at home allowed parents to perform other duties |  |  |  |  |  | √ |
| **4.3 Financing** |  |  |  |  |  |  |
| Belief that KMC cut down hospital bills due to early discharge | √ |  |  |  |  |  |
| Belief that kangaroo mother care was cheaper than incubator care | √ |  |  |  |  | √ |
| Parents more likely to stay if services were free | √ |  |  |  |  |  |
| Lower costs for health system |  |  |  | √ |  |  |
| Lowering hospital costs to families |  |  |  | √ |  |  |
| **5. Cultural Factors** |  |  |  |  |  |  |
| **5.1 Traditional newborn care** |  |  |  |  |  |  |
| Some HCWs advised mothers to delay bathing so infant would not get cold |  | √ |  |  |  |  |
| Type of wrap: customised |  |  |  |  | √ |  |
| **5.2 Traditional mindset** |  |  |  |  |  |  |
| Country-specific beliefs or practices |  |  | √ |  |  |  |
| Mother–infant confinement |  |  |  |  | √ |  |
| **5.3 Gender Roles** |  |  |  |  |  |  |
| Societal acceptance of paternal involvement | √ |  |  |  |  |  |
| Normalization of paternal involved in child care | √ |  |  |  |  |  |
| Male involvement |  |  |  |  | √ |  |
| Gender equality |  |  |  |  |  | √ |
