# Supplementary material for: What influences the implementation of kangaroo mother care? An umbrella review
Source: BMC Pregnancy Childbirth. 2022 Nov 18;22:851. doi: 10.1186/s12884-022-05163-3 (PMC9675107; doi:10.1186/s12884-022-05163-3)
Supplement: Supplementary file 4 — Additional file 4. Result of the quality appraisal of included studies. [file 12884_2022_5163_MOESM4_ESM.docx]

**Additional file4: Result of the quality appraisal of included studies**

| **Questions** | **Smith**  **et al. [27]** | **Chan**  **et al. [28]** | **Seidman**  **et al. [38]** | **Kinshella**  **et al. [29]** | **Mathias**  **et al. [39]** | **Chan**  **et al. [40]** |
| --- | --- | --- | --- | --- | --- | --- |
| 1. Is the review question clearly and explicitly stated? | Yes | Yes | Yes | Yes | Yes | Yes |
| 2. Were the inclusion criteria appropriate for the review question? | Yes | Yes | Yes | Yes | Yes | Yes |
| 3. Was the search strategy appropriate? | Yes | Yes | Yes | Yes | Yes | Yes |
| 4. Were the sources and resources used to search for studies adequate? | Yes | Yes | Yes | Yes | Yes | Yes |
| 5. Were the criteria for appraising studies appropriate? | Yes | Yes | No | Yes | No | Yes |
| 6. Was critical appraisal conducted by two or more reviewers independently? | Yes | Yes | No | Yes | No | Yes |
| 7. Were there methods to minimize errors in data extraction? | Yes | Yes | Yes | Yes | Yes | Yes |
| 8. Were the methods used to combine studies appropriate? | Yes | Yes | Yes | Yes | Yes | Yes |
| 9. Was the likelihood of publication bias assessed? | No | No | No | No | No | No |
| 10. Were recommendations for policy and/or practice supported by the reported data? | Yes | Yes | Yes | Yes | Yes | Yes |
| 11. Were the specific directives for new research appropriate? | Yes | Yes | Yes | Yes | Yes | Yes |
