# Supplementary material for: What influences the implementation of kangaroo mother care? An umbrella review
Source: BMC Pregnancy Childbirth. 2022 Nov 18;22:851. doi: 10.1186/s12884-022-05163-3 (PMC9675107; doi:10.1186/s12884-022-05163-3)
Supplement: Supplementary file 5 — Additional file 5. Risk of Bias analysis using ROBIS tool. [file 12884_2022_5163_MOESM5_ESM.docx]

**Additional file 5: Risk of Bias analysis using ROBIS tool.**

| **Article / Item Evaluated** | **Eligibility criteria of the studies** | **Identification and selection of the studies** | **Data collection and results evaluation** | **Findings synthesis** | **Risk of Bias Final Result** |
| --- | --- | --- | --- | --- | --- |
| Smith et al. [27] | Unclear | Low | Low | High | High Risk of Bias |
| Chan et al. [28] | Unclear | low | low | High | High Risk of Bias |
| Seidman et al.[38] | Low | low | High | High | High Risk of Bias |
| Kinshella et al.[29] | low | Low | Low | High | Unclear Risk of Bias |
| Mathias et al. [39] | low | low | low | High | Unclear Risk of Bias |
| Chan et al.[40] | Unclear | low | High | High | High Risk of Bias |
